# Determination of Geosmin and 2-Methylisoborneol and Associated Microbial Composition in Rainbow Trout Aquaculture Systems for Human Consumption

**DOI:** 10.3390/foods14142517

**Published:** 2025-07-18

**Authors:** Juan José Córdoba-Granados, Almudena V. Merchán, Carlos Moraga, Paula Tejero, Alberto Martín, María José Benito

**Affiliations:** 1 Carretera Cañada de la Loba (CA-3102) PK 3, Apdo. Correos, 589, 11590 Jerez de la Frontera, Cádiz, Spain; juanj.cordoba@juntadeandalucia.es; 2 Nutrición y Bromatología, Escuela de Ingenierías Agrarias, Universidad de Extremadura, Avd. Adolfo Suárez s/n, 06007 Badajoz, Spain; paulatc@unex.es (P.T.); amartin@unex.es (A.M.); mjbenito@unex.es (M.J.B.); 3 Instituto Universitario de Investigación en Recursos Agrarios (INURA), Universidad de Extremadura, Avd. de la Investigación, 06006 Badajoz, Spain; 4 Centro de Investigaciones Científicas de Extremadura (CICYTEX), Crta. A-V, Km 372, 06187 Guadajira, Badajoz, Spain; carlos.moraga@juntaex.es

**Keywords:** geosmin, 2-MIB, temperature, off-flavours, rainbow trout, aquaculture system, cyanobacteria

## Abstract

This study investigated the seasonal and spatial dynamics of off-flavour compounds—geosmin and 2-methylisoborneol (2-MIB)—in an intensive rainbow trout (*Oncorhynchus mykiss*) aquaculture system for human consumption in western Spain. Weekly water and fish flesh samples were collected over a 12-month period from three farms supplied by the River Tormes. Physicochemical parameters, determination of geosmin and 2-MIB by SPME-GC-MS, microbial counts, and microbial community composition were assessed alongside volatile compound concentrations. Geosmin and 2-MIB showed marked seasonal variation, with peak levels in water and fish flesh during spring and summer, correlating positively with temperature. Geosmin accumulation in fish was highest in the downstream farm, suggesting cumulative exposure effects. In contrast, 2-MIB was detected only in water and at lower concentrations. Microbial analyses revealed high bacterial and fungal diversity, including cyanobacterial taxa such as *Phormidium setchellianum* and *Pseudoanabaena minima*, known producers of geosmin and 2-MIB. These findings highlight the importance of water microbiota and environmental conditions in off-flavour development. Managing cyanobacterial populations and monitoring spatial-temporal variability are essential to mitigate the development of earthy or musty flavours and economic losses in aquaculture systems.

## 1. Introduction

Rainbow trout (*Oncorhynchus mykiss*) is a globally significant species in aquaculture, valued for its rapid growth and high nutritional quality [1]. According to the Food and Agriculture Organization [2], global production exceeds 1 million tonnes annually, underscoring its economic importance [3]. However, various factors affect both production and marketability, often leading to significant economic losses for producers. One of the most prominent issues in aquaculture is the presence of “muddy” off-flavours in fish, caused by the accumulation of volatile metabolites that negatively impact consumer perception and market prices [4]. These earthy and musty taints are primarily associated with geosmin (trans-1,10-dimethyl-trans-9-decalol) and 2-methylisoborneol (2-MIB) (exo-1,2,7,7-tetramethylbicyclo[2.2.1]heptan-2-ol) [5,6,7].

Even at low concentrations, these compounds can be absorbed by fish and accumulate in lipid-rich tissues [8], resulting in unpleasant taste and odour in both water [9] and fish [10]. Furthermore, both compounds can be perceived by human senses at extremely low concentrations (below 1 ng/g) [11,12]. While not hazardous to human health, geosmin and 2-MIB are highly resistant to conventional water treatment processes such as coagulation and sedimentation [13], making them a persistent problem in aquaculture systems.

The production of the secondary metabolites 2-MIB and geosmin is influenced by environmental factors, particularly phosphorus, nitrogen, and temperature [14]. In response, aquaculture facilities frequently employ oxidative processes to remove these off-flavour compounds from water. Treatments such as ozone, hydrogen peroxide, and potassium permanganate have demonstrated efficacy in decontaminating water by oxidising geosmin and 2-MIB [15,16]. Monitoring the physico-chemical parameters of water in aquaculture systems is essential for controlling the production and accumulation of geosmin and 2-MIB, as these factors directly influence the growth of odour-producing microorganisms and the uptake of these compounds by fish [17].

Geosmin is a sesquiterpene-derived alcohol produced by various microorganisms [8,18,19,20,21]. On the other hand, 2-MIB, a methylated monoterpene, is similarly produced by actinomycetes and cyanobacteria [22,23]. Microorganisms play a critical role in aquaculture systems, influencing water quality and fish health. Bacteria, which form a major component of aquatic ecosystems, affect not only the water but also the internal microbiota of fish, which plays a crucial role in digestion and immune response [24,25]. In the context of aquaculture, monitoring microbial populations is essential not only to ensure the health of the fish but also to prevent contamination that could pose risks to human health. Among the microbial communities in aquaculture, cyanobacteria are particularly noteworthy for their role in producing geosmin and MIB, the primary compounds responsible for off-flavours in fish. For instance, some *cyanobacteria* are nitrogen-fixing prokaryotes and have a competitive advantage over other members of the phytoplankton community, particularly in nutrient-limited environments [26]. Likewise, cyanobacterial species such as *Anabaena*, *Lyngbya*, *Oscillatoria*, *Phormidium*, and *Pseudanabaena* are well-documented producers of geosmin and MIB in freshwater systems [27,28,29]. Shen et al. [28] found that warm temperature favours cyanobacterial growth and increases the production of odour compounds. Cyanobacteria generally thrive at higher temperatures compared to eukaryotic algae, leading to seasonal blooms that contribute to the accumulation of geosmin and MIB in aquaculture waters [30,31]. Certain benthic cyanobacteria, such as *Phormidium setchellianum*, grow on substrates like rocks and the bottoms of water bodies, making them more likely to persist and proliferate in specific areas [32].

The aim of this study was to conduct comprehensive monitoring of the production dynamics of the off-flavour compounds geosmin and 2-MIB in three Spanish rainbow trout farms used for human consumption. Unlike previous studies [5,17], which focused solely on water monitoring, our approach included quantifying geosmin and 2-MIB in both water and fish flesh, as well as analysing key physicochemical parameters. Additionally, we characterised the temporal variation in microbial community composition to identify potential correlations between microbial dynamics, particularly those of geosmin- and 2-MIB-producing cyanobacteria, and the observed fluctuations in off-flavour compound concentrations. This integrative approach enhances our understanding of the biotic and environmental factors that contribute to off-flavour development in intensive aquaculture systems.

## 2. Materials and Methods

### 2.1. Sampling

From May 2023 to April 2024, rainbow trout and water samples were collected weekly from three fish farms located in Salamanca, Spain. These farms are supplied with water from the River Tormes and are situated on open land. As for the surface area of each farm, Farm 1 had an approximate surface area of 20,000 m^2^ and 31 ponds, Farm 2 had an approximate surface area of 10,000 m^2^ and 24 ponds, and Farm 3 had an approximate surface area of 13,000 m^2^ and 45 ponds. The water intake to three farms was 200 m^3^/s, with an oxygen level of 7 mg/L. In each farm, the water was treated by filters (which removed large particles) and settling tanks. The composition of the trout feed was the same on all farms and was mainly formulated with proteins and oils (from both fish and plants) as well as vegetable carbohydrates.

The fish were kept in the purification basins for 24–48 h without feeding before slaughter. The fishes were slaughtered on site and allowed to bleed. Each week, ten fish were randomly selected from each farm for flesh sampling and subsequent analysis. In parallel, ten water samples per farm were collected in 250 mL bottles, which were completely filled and immediately sealed to prevent headspace and potential compound loss. All samples were transported weekly under refrigerated conditions to the Agricultural Engineering School at the University of Extremadura for further analysis.

### 2.2. Determination of Physico-Chemical Parameters in Water Samples

Prior to sample collection, physico-chemical parameters (temperature (°C), oxygen (mg/L), CO_2_ (mg/L), pH, conductivity (µS/cm at 20 °C) and suspended matter (mg/L) of the water were determined during sampling. For nutrient measurements, the concentrations of ammonium (mg/L), total phosphorus (mg/L), total nitrogen (mg/L), nitrites (mg/L), nitrates (mg/L) and chemical oxygen demand (COD) of the water were determined during sampling. All water quality parameters were measured according to methods from APHA [33] and HACH [34] as previously described by Davidson et al. [35].

### 2.3. Geosmin and 2-MIB Determination in Water and Flesh Samples

For each water sample, triplicate subsamples of 3 mL of water were transferred to 15 mL glass bottles to which were added 1 g of NaCl (to increase the volatility of the taste and odour compounds). The glass bottles were capped with a silicone PTFE seal and kept at 4 °C until analysis. For each flesh sample, each fish was weighed and skinned. Approximately 10 g of flesh was minced and mixed with 50% cold Milli-Q water. This mixture was then homogenised for 30 s at 13,500 rpm using an Omnimixer. Then, 3 g of the resulting mixture and 1 g of salt were added to glass bottles, which were then stoppered until analysis.

To determine the concentration of geosmin and 2-MIB, a solid-phase microextraction (SPME) was used with a 2 cm 50/30 μm StableFlex fibre (part number 57348-U, Sigma Aldrich, St. Louis, MO, USA) in a manual fibre holder (part number 57330-U, Sigma Aldrich, St. Louis, MO, USA). The NaCl water samples were placed in a 60 °C water bath with a magnetic stirrer. The SPME fibre was exposed to headspace for 30 min to collect the volatile compounds. After absorption, the volatile compounds in the fibre were reabsorbed for 3 min at 260 °C in the injector of the gas chromatograph (GC-MS). The GC column had a diameter of 30 m × 0.25 mm, a film thickness of 0.25 mm, Rtx-5MS (Restek, Bellefonte, PA, USA), and 1 mL/min constant helium flux. The programme temperature was set at 45 °C for 3 min, increasing 30 °C/min constantly from there to 250 °C. Transfer line temperature and ion source temperature were 275 °C and 200 °C, respectively, with an exploration range of *m*/*z* 30–200. Geosmin and 2-MIB detection was estimated from fragments *m*/*z* 112 and 95, respectively.

Their identity was confirmed by comparing their mass spectra with those in a commercial database (National Institute of Standards and Technology, NIST), as well as by comparing their retention times with those of authentic standards. The MSD ChemStation software programme (version E.02.00, Agilent Technologies, Santa Clara, CA, USA) was used for data analysis. The relationship between peak area and concentration was linear, and the slopes of the resulting calibration curves were used to convert peak areas to concentrations. Linearity and detection limit of geosmin and 2-MIB were set using a standard of both compounds (10–100 ng/L) in water samples. To achieve absolute quantification, known quantities of geosmin and MIB (0, 1, 5 and 25 μg/kg) were added to selected fish flesh during homogenisation. To determine the reproducibility of the method, four flesh samples were taken from each of three fish and analysed. The detection threshold for geosmin in fish flesh was established at 10 ng/L in water, based on the sensory evaluation findings reported by Petersen et al. [17], which determined this concentration as the minimum level at which geosmin becomes perceptible in fish flesh.

### 2.4. Microbial Counts in Water Samples

For the microbial counts, water samples were collected prior to sample collection from the farms. Different microbial groups were analysed using selective culture media: Plate Count Agar (PCA) (Condalab, Madrid, Spain) for mesophilic aerobic bacteria, Violet Red Bile Glucose Agar (VRBG) (Condalab, Spain) for enterobacteria, Violet Red Bile Lactose Agar (VRBA) (Condalab, Spain) for total coliforms, Tryptone Bile X-glucuronide (TBX) (Condalab, Spain) for *Escherichia coli*, Slanetz–Bartley agar (Condalab, Spain) for faecal streptococci and Potato Dextrose Agar (PDA) (Condalab, Spain) for yeast. Microbial counts were expressed as log CFU/100 mL and were determined during sampling. Additional media were used to isolate specific microbial groups: BG-11 medium (Gibco, Waltham, MA, USA) for cyanobacteria. The biochemical oxygen demand (BOD_5_) was determined following the Standard Methods for the Examination of Water and Wastewater [36], by incubating the samples for 5 days at 20 °C in the dark and measuring dissolved oxygen before and after incubation.

### 2.5. Isolation and Identification of Bacteria, Yeast, Cyanobacteria and Algae

To obtain genomic DNA from bacteria and yeast isolates, 1 mL of each isolated culture, in Brain Heart Infusion broth (BHI, Condalab, Spain) for bacteria and Yeast Extract Peptone Dextrose broth (YPD, Condalab, Spain) for yeast, was collected by centrifugation at 10,000× *g* for 5 min at 4 °C. The pellet was suspended in lysis buffer and disrupted with 400 to 600 μm silica grinding beads in a 1600 MiniG homogenizer (SPEX SamplePrep, Metuchen, NJ, USA) at 1500 rpm for 5 min. Genomic DNA was extracted using the GeneJET Genomic DNA Purification Kit (Thermo Fisher Scientific, Waltham, MA, USA) according to the manufacturer’s instructions. For cyanobacteria and microalgae isolates, the DNA extraction was performed using the DNeasy^®^ Plant Mini Kit (QIAGEN^©^, Hilden, Germany) according to the manufacturer’s instructions, with the addition of an overnight proteinase K incubation at 56 °C for cyanobacterial cultures. The amount of DNA was determined by using a NanoDrop 2000 spectrophotometer (Thermo Fisher Scientific, USA), and the concentration was adjusted to 10 ng/μL for PCR reactions.

The isolates were sequenced with the following primers, using PCR conditions as described by the respective authors cited below. The bacterial isolates were identified at the species level by sequencing the 16S rRNA gene using 27F (5′-AGAGTTTGATCCTGGCTCAG-3′) and 1492R (5′-GGTTACCTTGTTACGACTT-3′) primers targeting V1–V9 regions [37]. For yeast and algae, the internal transcribed spacer ITS1/ITS2-5.8 S rDNA was amplified using the primer pairs ITS1/ITS4 (ITS1: 5′-CTTGGTCATTTAGAGGAAGTAA-3; ITS4: 5′-TCCTCCGCTTATTGATATGC-3′) [38]. For cyanobacteria, the 16S-23S rRNA gene ITS region was amplified using the primer pairs 27F (5′-AGAGTTTGATCCTGGCTCAG-3′) and 23S30R (5′-CTTCGCCTCTGTGTGCCTAGGT-3′) according to Jeong et al. [39]. For amplification, 10 μL of extracted DNA were added as a template in 50 μL reaction mixtures containing 50 pmol of primers, 500 mM of each dNTP, 0.1 vol of 10X PCR buffer (500 mM 23 Tris–HCl, pH 9.2, 140 mM (NH_4_)_2_SO_4_, 22.5 mM MgCl_2_), and 1 U Taq polymerase (Biotools B&M Labs, S.A., Madrid, Spain). PCR products were sent to the sequencing unit of the University of Extremadura (Service of Techniques Applied to Bioscience, STAB). The sequences were analysed with the software BioEdit Sequence Alignment, version 7.0.5.3 (Tom Hall) and compared with the EMBL/GenBank database using the BLAST algorithm (version 1.4.0). The isolates were confirmed based on the highest identity score (highest sequence homology).

### 2.6. Statistical Analysis

Statistical analysis was performed using the SPSS Statistics software package (version 22, IBM Corp., Armonk, NY, USA). One-way analysis of variance (ANOVA) was used to study the data. The mean values were separated for comparison using Tukey’s honestly significant difference test (HSD) at a significance level of *p* ≤ 0.05. Pearson’s correlation coefficient was used to assess correlations between the different parameters. Statistical significance was set at a *p*-value of <0.05. A principal component analysis (PCA) was also performed to obtain an overview of the relationship between the studied parameters. Data visualisations for the correlation heatmap and Venn diagram were generated using Python (v3.12) with the pandas (v2.2.2), numpy (v1.26.4), seaborn (v0.13.2), matplotlib (v3.8.4) and matplotlib-venn (v0.11.10) libraries.

## 3. Results

### 3.1. Physico—Chemical Parameters of Aquaculture Facilities

A weekly monitoring of physico-chemical parameters of water samples was performed from May 2023 to April 2024, including measurements of temperature and gas concentrations (dissolved oxygen and carbon dioxide), water chemistry and quality (pH, conductivity and chemical oxygen demand (COD)), nutrient levels (ammonia, phosphate, nitrogen, nitrite and nitrate) and physical properties such as suspended matter. The global results have been summarised in Table 1.

The results show that the water temperature oscillated between 14 and 7 °C with an average of 11.51 ± 1.13 °C, demonstrating significant seasonal variation. The pH values remained relatively stable around neutrality, averaging 6.54 ± 0.44 with little change over time. The levels of dissolved oxygen exhibited considerable variability, with an average of 8.71 ± 2.7 mg/L indicative of changes in water flow and biological activity. Carbon dioxide levels were found to be low overall, averaging at 6.54 mg/L, with a range from 3.2 to 10.6 mg/L. This finding reflects stable respiration and minimal organic decomposition in the water. The nutrient concentrations were generally low, with ammonia averaging at 0.23 ± 0.09 mg/L and total phosphate at 0.16 ± 0.20 mg/L, suggesting good nutrient management. The nitrate levels were moderate (0.43 ± 0.11 mg/L), while the nitrite concentrations were minimal (0.01 ± 0.005 mg/L). The suspended matter remained at a low level (2.35 ± 0.46 mg/L), and the chemical oxygen demand averaged at 13.25 ± 4.92 mg/L. These findings indicate favourable conditions for rainbow trout farming throughout the monitoring period.

**Figure 1:**
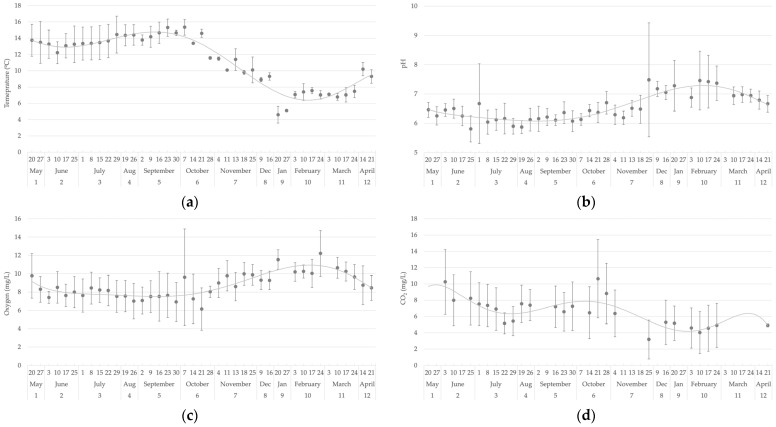
Evolution over the year of (**a**) temperature, (**b**) pH, (**c**) dissolved oxygen (mg/L), and (**d**) dissolved carbon dioxide (mg/L).

Figure 1 shows the evolution of the water parameters analysed throughout the weekly period. The temperature (Figure 1a) exhibited a maximum value of 15.3 °C in September and October and a minimum value of 4.6 °C in January. The pH levels (Figure 1b) exhibited fluctuations between 5.8 and 7.5, with notable peaks observed during the winter months. Oxygen concentrations (Figure 1c) displayed some variability, particularly during the colder months when higher values were observed. The CO_2_ levels (Figure 1d) showed their highest concentrations during the spring and autumn periods.

**Figure 2:**
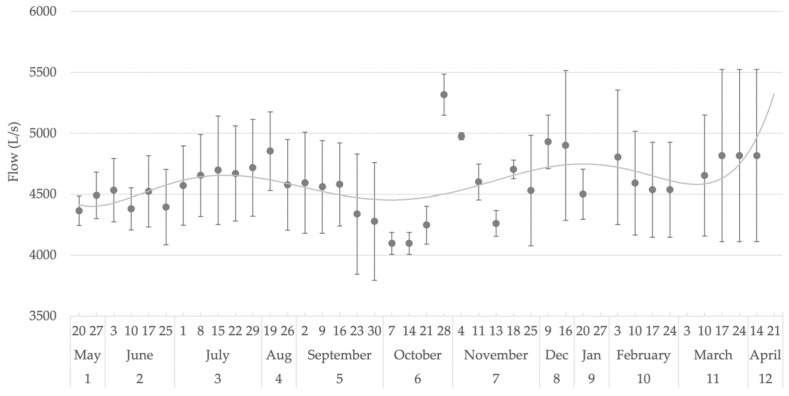
Annual flow dynamics in water systems.

Regarding the flow rate of the water systems, Figure 2 shows the annual flow dynamics during the year. The monthly average flow rate ranged from a minimum of around 4427 L/s in May to a maximum of approximately 4915 L/s in December. Higher flow rates were generally observed during the summer months (June to August), with averages consistently exceeding 4500 L/s, indicating greater water availability during this period. In contrast, lower values were concentrated in autumn, particularly in October. Relatively stable flow rates were observed in transitional seasons such as spring (March and April), averaging close to 4800 L/s.

### 3.2. Geosmin and 2-MIB Dynamics in Water Farms and Rainbow Trout Flesh

**Figure 3:**
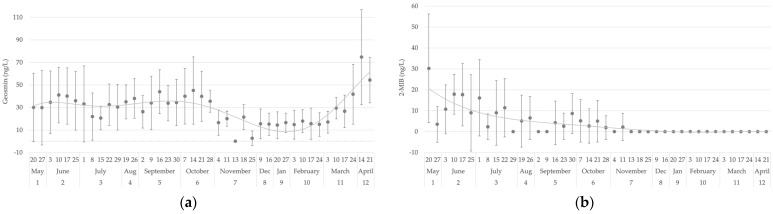
Evolution over the year of (**a**) geosmin and (**b**) 2-MIB measured in ng/L in water samples.

In this section, the results of geosmin and 2-MIB concentrations in both water and trout flesh samples throughout the study period are presented. The baseline threshold for each compound was established at 10 ng/L and 0.1 µg/L in water and flesh samples, respectively. The analysis of geosmin and 2-MIB concentrations in water samples (Figure 3) revealed a marked seasonal variation. Geosmin levels (Figure 3a) exhibited a peak in April, reaching 74.7 ng/L, and generally fluctuated between 14 and 45 ng/L during the warmer months, with the lowest concentrations recorded in winter (November to January), when values often fell to zero. In a similar manner, concentrations of 2-MIB (see Figure 3b) demonstrated a maximum in May, with values reaching 30 ng/L. However, these concentrations also underwent a substantial decline during the colder months.

**Figure 4:**
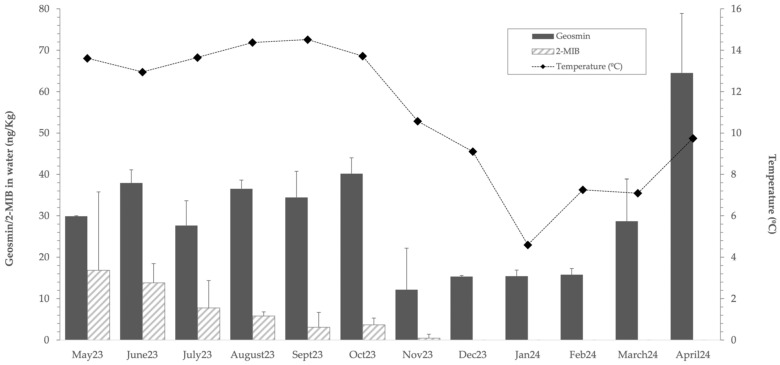
SPME–GC–MS analysis of geosmin and 2-MIB levels (ng/kg) in water and the monthly water temperature profile (°C).

In consideration of the potential correlation between geosmin and 2-MIB concentrations with the temperature profile over the course of the year, Figure 4 demonstrates that elevated levels of volatile compounds are observed when the temperature ranges between 10 and 15 °C. Furthermore, a positive correlation (*p* < 0.05) with higher concentrations of these compounds is identified, indicating that at elevated temperatures, the concentration of each compound tends to increase.

**Figure 5:**
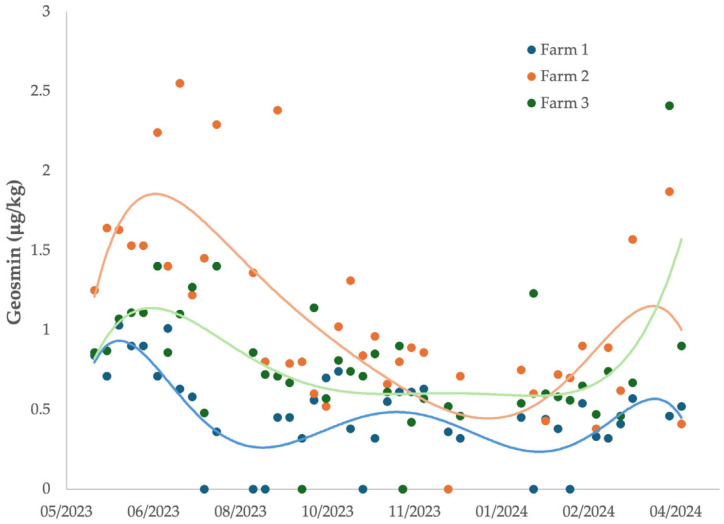
Monthly variation in geosmin concentration in rainbow trout flesh in the different farms.

The distribution of geosmin concentrations in trout flesh varied notably between farms (Figure 5), highlighting spatial heterogeneity in its accumulation and persistence within the aquaculture system. The highest concentrations of geosmin in the flesh were found in Farm 2, which is located in the middle of the facility. This can be attributed to the accumulation of sludge at the bottom of this farm, which allows geosmin to accumulate and geosmin-producing microorganisms to proliferate. Conversely, 2-MIB was not detected in any of the samples taken from the flesh at any point during the measurement period.

Looking at each farm, it was observed that the trout from the farm with the highest geosmin levels were found in Farms 2 and 3 and the lowest in Farm 1. This may again be due to Farm 1 being the first system upstream and therefore the least contaminated. Farms 2 and 3 were the systems with the highest amount of geosmin in the water and therefore the highest accumulation in the trout flesh.

Correlation analysis (Table 2) revealed a statistically significant association (*p* < 0.05) between geosmin concentrations in the water—both upstream and downstream—and its accumulation in trout flesh, thereby supporting the hypothesis of a direct transfer and bioaccumulation of this off-flavour compound from the water to the fish. In contrast, 2-MIB concentrations in the water exhibited no statistically significant correlations with its levels in flesh (*p* > 0.05), suggesting a less consistent or efficient transfer mechanism for this compound. Furthermore, a significant positive correlation was observed between water temperature and 2-MIB concentrations at the upstream site (*p* < 0.05), suggesting a potential influence of thermal conditions on its production or persistence. However, no significant correlations were detected between 2-MIB in water and 2-MIB concentrations in the flesh. These results emphasise the central role of waterborne geosmin as a predictor of its presence in trout flesh, while pointing to a weaker and more complex relationship in the case of 2-MIB.

The application of correlation analysis yielded several significant associations between the physico-chemical parameters of the water and the concentrations of geosmin and 2-MIB (Table 3). At the upstream location, a robust negative correlation was observed between water temperature and dissolved oxygen levels (*p* < 0.01). Additionally, a moderate positive correlation was identified between water temperature and CO_2_ levels (*p* < 0.01). These findings imply that elevated water temperatures are associated with diminished oxygen availability and augmented CO_2_ levels. Conversely, 2-MIB exhibited no substantial correlations with the physico-chemical at the upstream location. Downstream, geosmin exhibited a significant negative correlation with both pH (*p* < 0.01) and dissolved oxygen (*p* < 0.05), thereby reinforcing its apparent sensitivity to these environmental conditions. Furthermore, a negative correlation was observed between 2-MIB concentration downstream and pH (*p* < 0.01), suggesting a potential influence of water acidity on its presence. Furthermore, a positive correlation was observed between this compound and CO_2_ levels (*p* < 0.05). No other significant associations were observed for this compound. Conversely, a negative correlation was found between flow (L/s), temperature, geosmin and 2-MIB levels either upstream or downstream, but no statistical associations were observed between these parameters. Collectively, these results emphasise the intricate and location-specific relationships between water quality parameters and the occurrence of off-flavour compounds in aquaculture systems.

**Figure 6:**
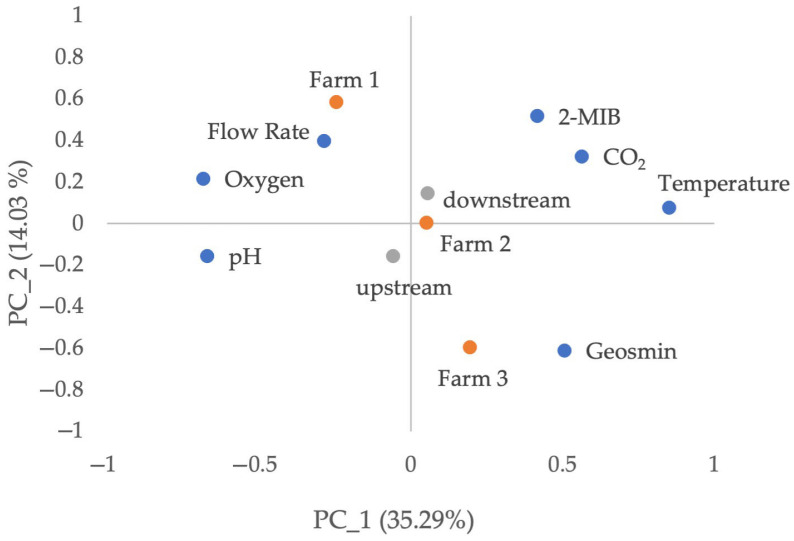
Principal component analysis (PCA) of the location (upstream or downstream), physico-chemical parameters and farms evaluated in this study.

Figure 6 illustrates the principal component analysis (PCA) of the physico-chemical parameters, volatile compounds and locations of the measures. Despite the presence of geosmin and 2-MIB in the water samples, a clear association was observed between these compounds and Farm 3, with a weaker relationship noted to Farm 2. The prevailing temperature was identified as the pivotal parameter exerting influence on the observed pattern, with its escalation corresponding to augmented concentrations of geosmin and 2-MIB. Furthermore, an inverse relationship was observed between flow rate and geosmin levels, suggesting that an increase in flow rate results in a decrease in geosmin concentrations. This finding offers a potential explanation for the observation that Farm 1, which exhibited consistently higher flow rates, demonstrated lower geosmin concentrations. The water’s inlet (upstream) and outlet (downstream) locations demonstrated no substantial impact on geosmin levels. Furthermore, the oxygen concentration was found to be a contributing factor; higher levels of oxygen were observed to be correlated with a decrease in geosmin concentrations.

**Figure 7:**
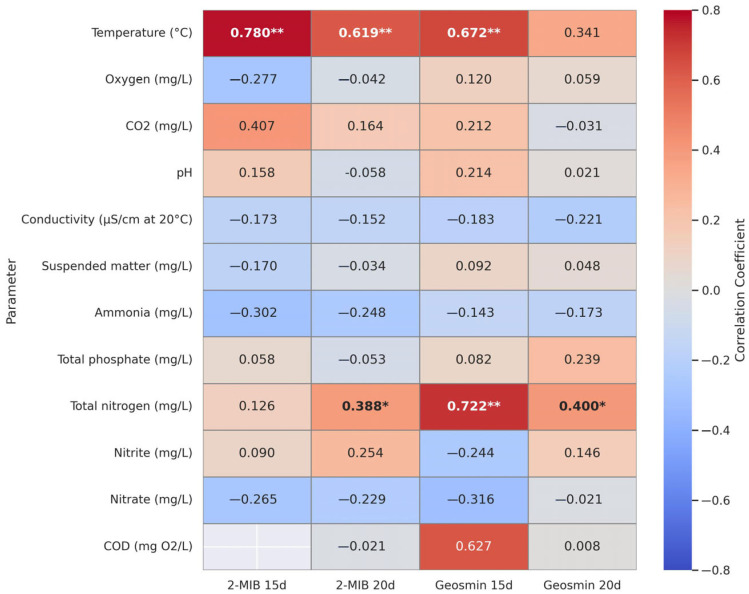
Heatmap showing the Pearson correlation coefficients between physicochemical parameters of aquaculture water and the concentrations of 2-MIB and geosmin at 15 and 20 days post-sampling. Numerical values within each cell represent the correlation coefficient. Asterisks indicate statistically significant correlations (* *p* < 0.05; ** *p* < 0.01).

In addition to the noticeable effect of temperature from day one, this study aimed to determine whether the compounds responsible for unpleasant odours and flavours increased or decreased immediately or over the course of several days. Therefore, the physicochemical parameters were correlated with the concentrations of geosmin and 2-MIB detected in the water after 15 and 20 days (Figure 7).

Statistically significant correlations were identified between specific physicochemical water parameters and geosmin and 2-MIB concentrations during the monitoring period. Water temperature exhibited significant positive associations with geosmin and 2-MIB concentrations at 15 and 20 days, respectively (*p* < 0.01), suggesting that this parameter may play a key role in the accumulation of off-flavour compounds in aquaculture systems. Additionally, total nitrogen showed significant correlations with geosmin at 15 days and 20 days (*p* < 0.01 and *p* < 0.05, respectively) and with 2-MIB at 20 days (*p* < 0.05), indicating a potential link between the organic load of the water and the presence of these compounds. However, no statistically significant correlations were observed with any of the other analysed parameters, including dissolved oxygen, carbon dioxide, pH, conductivity, suspended solids, ammonia, nitrites or nitrates.

### 3.3. Microbiological Analysis of Water Samples from the Aquaculture System

Microorganisms constitute a primary producer of geosmin and 2-MIB compounds within aquatic environments. In this context, a comprehensive analysis of the water microbiota has been carried out in samples from the different farms. A general microbiological analysis (Table 4) was performed in order to evaluate the cell concentration of each group of microorganisms in water. The presence of coliform microorganisms, including *Escherichia coli*, was not detected in certain water samples. However, the concentration of total coliforms attained a maximum value of 3.78 log CFU/100 mL in some samples, with a mean concentration of 3.21 log CFU/100 mL being recorded. Regarding the faecal streptococci, the values obtained showed a maximum of 2.8 log CFU/100 mL in certain samples (mean 2.08 log CFU/100 mL).

**Figure 8:**
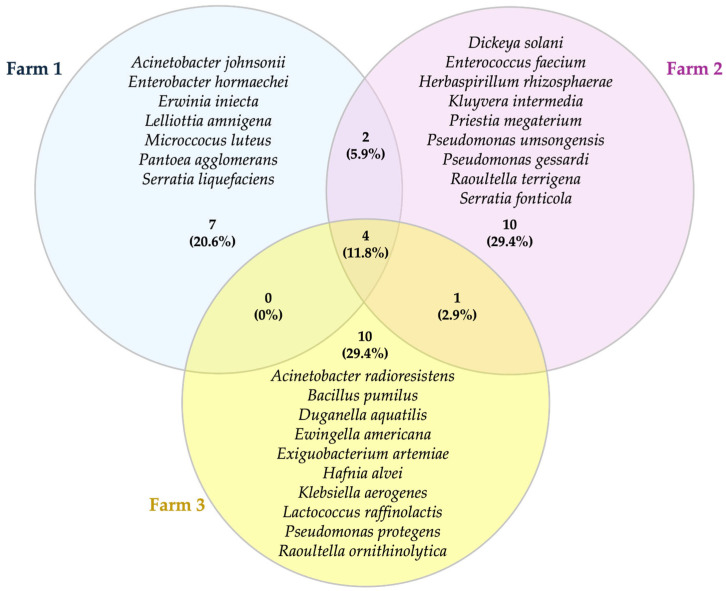
Venn diagram representing shared and unique species of bacteria between water samples from different farms. Numbers in brackets indicate the percentage of bacterial taxa belonging to each sample.

Amplicon sequencing of the 16S rRNA and ITS genes was performed on a total of 625 isolates to assess the different taxa present in the water samples. Microorganisms from 15 different families were isolated from water samples. Among the bacterial taxa, *Enterobacteriaceae* was the most prevalent family, followed by *Pseudomonadaceae* and *Bacillaceae*. As shown in the Venn diagram (Figure 8), the majority of the bacterial species were found in specific ponds, with only four species being common to the three sites (*Bacillus mycoides*, *B. safensis*, *B. cereus* and *Pseudomonas koreensis*). Two species were found in both Farm 1 and Farm 2, namely *Erwinia persicina* and *Pseudomonas fluorescens*, while Farm 2 and Farm 3 exhibited the presence of only one species, *Acinetobacter haemolyticus*. Conversely, no species was identified as being present in both Farm 1 and Farm 3.

Among fungal taxa, *Umbelopsidaceae* and *Saccharomycetaceae* were the most abundant families in the different farms, followed by *Sporidiobolaceae* and *Filobasidiaceae*. A total of eight fungal species were isolated and identified at the monitoring sites. Quantitative analysis revealed the highest species richness in Farm 3, where the following fungal species were recovered from water samples: *Cryptococcus randhawii*, *Pichia anomala*, *P. guilliermondii*, *Rhodotorula babjevae*, *R. mucilaginosa* and *Umbelopsis vinacea*. In contrast, Farm 1 exhibited a significantly reduced fungal diversity compared to Farm 3, with the isolation of only *U. isabelline* and *Kazachstania exigua*. Notably, no fungal species were detected in samples from Farm 2.

**Figure 9:**
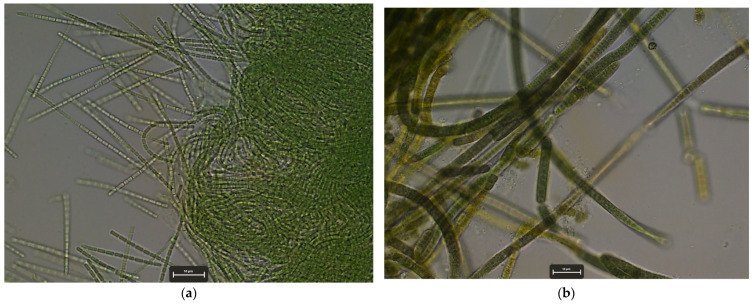
Cyanobacteria isolated from water samples. (**a**) *Pseudanabaena minima* and (**b**) *Phormidium setchellianum*.

In addition to the bacterial and fungal microbiota, the presence of other microorganisms capable of producing geosmin and 2-MIB, in particular cyanobacteria and algae, was also investigated. A total of 260 isolates were obtained from cyanobacteria. The identification of these isolates enabled the differentiation of two species of cyanobacteria, *Phormidium setchellianum* and *Pseudoanabaena minima* (Figure 9), in the three farms, with *Ph. setchellianum* being the most prevalent in the different samples. Furthermore, a total of 140 algae were isolated from the three farms and could be identified down to genus level as *Eudorina* sp., *Monoraphidium* sp., *Pandorina* sp. and *Desmodesmus* sp.

## 4. Discussion

Weekly monitoring of physicochemical parameters in three rainbow trout (*Oncorhynchus mykiss*) fish farms from May 2023 to April 2024 revealed generally stable and favourable water quality conditions for the intensive cultivation of this species. Water temperature exhibited expected seasonal fluctuations, remaining within a thermal range suitable for rainbow trout, promoting optimal metabolic activity and growth rates [40]. The pH remained close to neutrality, a critical factor for maintaining physiological homeostasis in aquatic organisms and minimising stress, thereby reducing susceptibility to disease [41]. Dissolved oxygen concentrations consistently exceeded the minimum recommended thresholds for salmonid species, particularly during colder months, when higher levels were observed. This trend aligns with the increased solubility of oxygen at lower temperatures and a potential reduction in the metabolic activity of aquatic organisms during winter [42]. Carbon dioxide (CO_2_) concentrations remained relatively low throughout the monitoring period, with peak values recorded in spring. This may be associated with increased respiratory activity of phytoplankton and microbial communities. Nutrient levels, including ammonia, total phosphate, and nitrate, remained within acceptable limits throughout the monitoring period, which is essential for maintaining water quality and preventing toxicity risks to fish [43]. Suspended solids concentrations were low, contributing to good water clarity and reducing the risk of gill irritation or mechanical stress in fish. Chemical oxygen demand (COD) values were moderate, suggesting a low presence of oxidisable pollutants and organic matter inputs. Additionally, water flow rates remained relatively stable during transitional seasons, particularly in March and April, indicating effective hydraulic management of the system.

Overall, these findings indicate that the physicochemical conditions across the three aquaculture farms were well-regulated and suitable for the cultivation of rainbow trout throughout the study period. Furthermore, the relatively low loads of nutrients and organic matter suggest a limited risk of excessive microbial proliferation. However, seasonal fluctuations in temperature and dissolved oxygen may influence microbial dynamics and the potential production of off-flavour compounds such as geosmin and 2-MIB [44,45].

Despite these fluctuations, the overall variation observed in temperature, dissolved oxygen, pH, CO_2_, and flow rate underscores the need for dynamic and seasonally adjusted water quality management, particularly in intensive aquaculture systems where even minor changes can significantly impact fish physiology, microbial activity, and potentially the formation of undesirable compounds such as geosmin and 2-MIB.

In this context, the results obtained throughout the study period revealed a clear seasonal variation in the concentrations of geosmin and 2-MIB in both water and trout flesh, with the highest peaks recorded during spring and summer. This pattern aligns with previous studies reporting increased production of off-flavour compounds during warmer months [44,46]. In contrast, during the winter months, concentrations of both compounds decreased significantly, in some cases reaching undetectable levels, reflecting a strong influence of ambient temperature on the production of these metabolites. The significant positive correlation between water temperature and the concentrations of geosmin and 2-MIB supports the hypothesis that elevated thermal conditions enhance the growth suitability of specific microorganisms responsible for the biosynthesis of these compounds, such as actinobacteria and cyanobacteria [47]. However, this relationship was more evident for concentrations in water than in fish flesh, suggesting that bioaccumulation in muscle tissue may be modulated by additional factors, including prolonged exposure, fish metabolism, or spatial positioning within the aquaculture system. Notably, geosmin concentrations in trout flesh varied significantly among farms, with the highest levels observed in Farm 2, located in the middle of the system. This suggests a cumulative effect in fish exposed to higher concentrations and longer durations of volatile compounds present in the water and sludge. The positive correlation between geosmin concentrations in water and its accumulation in flesh further supports this observation. In contrast, 2-MIB was detected at lower concentrations in water and was not detected in any of the flesh samples, reinforcing the importance of both concentration and exposure time in determining tissue accumulation. However, this study is limited by the absence of a sensory panel to validate the perception of off-flavours in fish flesh. While the quantification of geosmin and 2-MIB provides valuable data, the relationship between chemical concentration and sensory perception can be variable. Petersen et al. [17] demonstrated this by determining the sensory detection threshold for geosmin in rainbow trout flesh using a trained panel and establishing a threshold of 10 ng/L in water. Future research should incorporate sensory evaluations to better assess the impact of these compounds on consumers.

Finally, correlation analyses between the physicochemical parameters of the water and the presence of geosmin and 2-MIB also revealed complex, location-dependent relationships within the system. The observed correlations with total nitrogen levels suggest that nutrient enrichment possibly influences the structure of the microbial community and the presence of secondary metabolite producers. Elevated nitrogen levels contribute to eutrophication [46], favouring the proliferation of cyanobacteria and actinobacteria that produce off-flavour compounds. This nutrient-driven microbial activity is widely recognised as a key factor in the accumulation of geosmin and 2-MIB in aquaculture systems, especially when water exchange is limited and the organic load is high [28,46].

Taken together, these results highlight the importance of both spatial and seasonal factors in the dynamics of off-flavour compounds in intensive aquaculture systems, which may also be closely linked to the microbiological characteristics of these environments.

Microbiological analyses of the water revealed a diverse and complex microbial community, with notable variations in composition and abundance among the different farms. These findings are particularly relevant given the critical role played by certain microorganisms—especially bacteria, fungi and cyanobacteria—in the biosynthesis of off-flavour compounds such as geosmin and 2-MIB [48].

The quantification of mesophilic aerobic bacteria indicated generally high microbial amounts within the system. Enterobacteria and coliforms were present at relevant concentrations, although *Escherichia coli* was detected only sporadically, suggesting limited faecal contamination. Taxonomic analysis revealed a high prevalence of members of the family *Enterobacteriaceae*, followed by *Pseudomonadaceae* and *Bacillaceae* [49]. Regarding the fungal component, greater diversity was observed in Farm 3, which may be associated with higher organic amounts or more stable conditions for the growth of yeasts and moulds. Overall, this heterogeneity suggests differences in nutrient availability, pH, temperature, or biological interactions that either limit or promote the development of specific microbial communities.

However, the significant presence of photosynthetic microorganisms—particularly cyanobacteria—may provide an explanation for the production of geosmin and 2-MIB. Two cyanobacterial species, *Phormidium setchellianum* and *Pseudoanabaena minima*, were identified across all farms, of which *Ph. setchellianum* was the most frequently isolated and identified species. Both genera have previously been reported as producers of geosmin and/or 2-MIB [50], positioning them as potential direct sources of these compounds within the system. Additionally, planktonic microalgae belonging to different genera typically found in eutrophic systems—such as *Eudorina*, *Pandorina*, *Monoraphidium* and *Desmodesmus*—were also isolated. Although these genera have not been reported as producers of off-flavour compounds, their presence may indirectly influence the microbiota through nutrient competition or the production of secondary metabolites.

Overall, these results demonstrate a close relationship between the microbial composition of the water and the dynamics of volatile compounds (geosmin and 2-MIB) in rainbow trout aquaculture systems.

## 5. Conclusions

Overall, these results underscore the importance of both spatial and seasonal factors in the dynamics of off-flavour compounds within intensive rainbow trout aquaculture systems for human consumption. The correlation between geosmin concentrations in water, temperature, total nitrogen and its accumulation in trout flesh highlights the need for prioritised monitoring and control of this compound in the aquatic environment to prevent economic losses associated with diminished organoleptic quality of the final product.

The identification of cyanobacterial species with the potential to produce geosmin and 2-MIB, combined with previously reported concentrations of these compounds in water and fish flesh, suggests that managing algal and cyanobacterial populations could be an effective strategy to mitigate the development of earthy or musty flavours. The marked variability observed among farms further indicates the necessity of a site-specific approach to microbiological water quality management. This may include the implementation of targeted biofiltration, light and nutrient control, or even specific treatments such as depuration of fish in ozonated clean water, aimed at eliminating or limiting the growth of off-flavour-producing species without compromising the overall health of the aquaculture ecosystem. Moreover, identifying genes involved in geosmin and 2-MIB biosynthesis in cyanobacterial species could support the development of molecular tools for early detection and control, particularly under high-temperature seasons that favour their proliferation.

## Figures and Tables

**Table 1:** Descriptive statistics of physico-chemical parameters measured.

	N	Mean		SD	Min	Max
**Temperature (°C)**	312	11.51	±	1.13	4.60	15.34
**pH**	312	6.54	±	0.44	5.81	7.49
**Oxygen (mg/L)**	312	8.71	±	2.70	6.14	12.21
**CO_2_ (mg/L)**	312	6.54	±	2.06	3.18	10.63
**Conductivity (µS/cm at 20 °C)**	312	115	±	9.9	96	132
**Suspended matter (mg/L)**	312	2.35	±	0.46	1.80	3.50
**Ammonia (mg/L)**	312	0.23	±	0.09	0.12	0.49
**Total phosphate (mg/L)**	312	0.16	±	0.20	0.03	0.99
**Total nitrogen (mg/L)**	312	10.49	±	3.42	6.70	17.60
**Nitrite (mg/L)**	312	0.01	±	0.005	0.01	0.02
**Nitrate (mg/L)**	312	0.43	±	0.11	0.20	0.80
**COD (mg O_2_/L)**	312	13.25	±	4.92	7.34	21.30

**Table 2:** Correlation analysis between the off-flavour concentration in water and flesh and temperature.

Correlation	Temperature (°C)		Geosmin in Flesh (μg/kg)		2-MIB in Flesh (μg/kg)	
	Pearson	α	Pearson	α	Pearson	α
***Farm Upstream***						
**Geosmin (ng/L)**	0.330	0.043 (*) ^1^	0.392	0.012 (*)	−0.078	0.632
**2-MIB (ng/L)**	0.395	0.014 (*)	0.326	0.040 (*)	0.030	0.856
***Farm Downstream***						
**Geosmin (ng/L)**	0.435	0.005 (**)	0.473	0.002 (**)	−0.119	0.466
**2-MIB (ng/L)**	0.353	0.025 (*)	0.443	0.004 (**)	−0.081	0.619

^1^ (*): *p* < 0.05; (**): *p* < 0.01.

**Table 3:** Correlation analysis between physico-chemical parameters, temperature and off-flavour compounds in water samples.

Correlation	pH		Oxygen (mg/L)		CO_2_ (mg/L)		Flow (L/s)	
	Pearson	α	Pearson	α	Pearson	α	Pearson	α
***Farm Upstream***								
**Temperature (** **°** **C)**	−0.537	0.000 (**) ^1^	−0.656	0.000 (**)	0.471	0.000 (**)	−0.120	0.195
**Geosmin (ng/L)**	−0.228	0.012 (*)	−0.340	0.000 (**)	0.189	0.058	−0.121	0.188
**2-MIB (ng/L)**	−0.174	0.055	−0.02	0.830	0.149	0.136	−0.071	0.443
***Farm Downstream***								
**Temperature (** **°** **C)**	−0.487	0.000 (**)	−0.528	0.000 (**)	0.441	0.000 (**)	−0.133	0.083
**Geosmin (ng/L)**	−0.213	0.009 (**)	−0.197	0.015 (*)	−0.053	0.296	−0.121	0.104
**2-MIB (ng/L)**	−0.162	0.038 (*)	−0.019	0.419	0.179 (*)	0.03 (*)	−0.071	0.231

^1^ (*): *p* < 0.05; (**): *p* < 0.01.

**Table 4:** Descriptive statistics of the microbiological analysis of water microbiota. Microbial counts are expressed in log CFU/100 mL.

	N	Mean		St. Dev.	Min	Max
**Aerobic Mesophilic Bacteria**	312	4.53	±	1.27	3.54	5.60
**Total Enterobacteria**	312	4.21	±	1.31	2.70	5.20
**Total Coliforms**	312	3.21	±	1.15	2.41	3.78
***E. coli***	312	1.51	±	0.89	0.00	1.90
**Faecal Streptococci**	312	2.08	±	1.76	1.00	2.80
**Total Yeast**	312	2.62	±	1.07	2.00	3.60
**BOD_5_ (mg/L)**	312	1.05	±	0.81	0	3.90

## Data Availability

Data is contained within the article.

## Abbreviations

The following abbreviations are used in this manuscript:

2-MIB	2-methylisoborneol
